# High resolution assembly and characterization of genomes of Canadian isolates of *Salmonella* Enteritidis

**DOI:** 10.1186/1471-2164-15-713

**Published:** 2014-08-25

**Authors:** Dele Ogunremi, John Devenish, Kingsley Amoako, Hilary Kelly, Andrée Ann Dupras, Sebastien Belanger, Lin Ru Wang

**Affiliations:** Ottawa Laboratory Fallowfield, Canadian Food Inspection Agency, 3851 Fallowfield Road, Ottawa, Ontario K2H 8P9 Canada; Lethbridge Laboratory, Canadian Food Inspection Agency, Township Road 9-1, P. O. Box 640, Lethbridge, Alberta T1J 3Z4 Canada; Greater Toronto Area Laboratory, Canadian Food Inspection Agency, 2301 Midland Avenue, Scarborough, Ontario M1P 4R7 Canada

**Keywords:** *Salmonella* Enteritidis, Genomes, Core, Accessory, Single nucleotide polymorphism, Subtyping, Tracking

## Abstract

**Background:**

There is a need to characterize genomes of the foodborne pathogen, *Salmonella enterica* serovar Enteritidis (SE) and identify genetic information that could be ultimately deployed for differentiating strains of the organism, a need that is yet to be addressed mainly because of the high degree of clonality of the organism. In an effort to achieve the first characterization of the genomes of SE of Canadian origin, we carried out massively parallel sequencing of the nucleotide sequence of 11 SE isolates obtained from poultry production environments (n = 9), a clam and a chicken, assembled finished genomes and investigated diversity of the SE genome.

**Results:**

The median genome size was 4,678,683 bp. A total of 4,833 chromosomal genes defined the pan genome of our field SE isolates consisting of 4,600 genes present in all the genomes, i.e., core genome, and 233 genes absent in at least one genome (accessory genome). Genome diversity was demonstrable by the presence of 1,360 loci showing single nucleotide polymorphism (SNP) in the core genome which was used to portray the genetic distances by means of a phylogenetic tree for the SE isolates. The accessory genome consisted mostly of previously identified SE prophage sequences as well as two, apparently full- sized, novel prophages namely a 28 kb sequence provisionally designated as SE-OLF-10058 (3) prophage and a 43 kb sequence provisionally designated as SE-OLF-10012 prophage.

**Conclusions:**

The number of SNPs identified in the relatively large core genome of SE is a reflection of substantial diversity that could be exploited for strain differentiation as shown by the development of an informative phylogenetic tree. Prophage sequences can also be exploited for SE strain differentiation and lineage tracking. This work has laid the ground work for further studies to develop a readily adoptable laboratory test for the subtyping of SE.

**Electronic supplementary material:**

The online version of this article (doi:10.1186/1471-2164-15-713) contains supplementary material, which is available to authorized users.

## Background

*Salmonella* Enteritidis (SE) has emerged as the most commonly isolated serovar of foodborne *Salmonella* in humans over the last two decades [1–3]. SE belongs to a larger group of pathogens known as non-typhoidal *Salmonella* which ranked the most hazardous when a number of health indices were used to assess the 14 most burdensome foodborne bacteria, viruses and parasites causing diseases in humans [4, 5]. In Canada, the proportion of human salmonellosis caused by SE increased from 13% in 2003 to 38% in 2010 [6]. In the US, an outbreak of SE in 2010 resulted in an estimated 1,939 human illnesses (http://www.cdc.gov/salmonella/enteritidis/) and the largest egg recall in the country’s history involving over 500 million shell eggs (http://www.fda.gov/Safety/Recalls/MajorProductRecalls/ucm223522.htm [7]).

Early comparative analysis of SE with the serovar Typhimurium, the latter being one of the best studied *Salmonella* serovars because of its enduring importance as a human pathogen and wide host range among vertebrates, has led to the identification of metabolic pathways and virulence genes for SE. The application of DNA methodologies especially the adoption of DNA-DNA hybridization technique as the reference method of establishing relationships among organisms [8, 9] provided insight on the genetic relatedness among many *Salmonella* serovars [10]. Consequently, the majority of *Salmonella* serovars *-* 2,587 at the last count [11] - including serovar Enteritidis, were classified into a single species, namely: *S. enterica.* The remaining 23 known serovars, which are typically but not exclusively isolated from cold blooded animals, belong to the second species known as *S. bongori* [12] *.* This re-designation of a majority of *Salmonella* organisms previously known by their species designation into a single species because of observed genetic similarities conflicted with historical and behavioural differences observed in their host ranges. Now commonly described by their serovar designations, organisms belonging to the *S. enterica* species fall into three broad groups based on the ability to either infect only a single type of vertebrate host (e.g., serovars Typhi and Pullorum of humans and poultry, respectively)*,* or a limited number of hosts usually including humans (e.g., serovars Dublin and Choleraesuis) or an extensive host range (e.g., serovars Enteritidis and Typhimurium). Other notable differences were observed among serovars with similar host range patterns including cultural growth patterns and biochemical test results in the laboratory. The ensuing conundrum was that observed genetic similarities among serovars was at variance with considerable behavioural differences and this has now led to an urgent need to develop a robust subspecies and sub-serovar level classification [13]. To compound the situation, it is clear that isolates of the serovar Enteritidis show an even more remarkable and striking genetic similarity with one another to the extent that existing phenotypic and genotypic bacterial typing tools have proven inadequate to assess their degree of relatednesss [14–16]. Despite these reported similarities a number of other studies have identified differences among isolates of Enteritidis in animal infection trials, cell invasion assays, growth rates and ability to contaminate and survive within eggs [17–19]. The above underscores the need for a much deeper insight into the biology of the SE which has become accentuated by its new prominence as a foodborne pathogen of humans. The increasing success of a pathogen that displays as much clonality as SE is intriguing and not easily explained from a biological perspective. An effective immune response of a vertebrate host against clonal bacteria would almost certainly protect the host against further exposure to the same or similar strains. It is therefore to the advantage of the pathogen to have a capacity and means of evading the host’s immune system by changing its antigenic properties. Thus, clonality of a pathogen may ordinarily be seen as a disadvantage from an evolutionary viewpoint. Yet, this has not impeded the success of SE as a thriving pathogen. From the perspective of a food microbiology laboratory, the clonality of SE has made it extremely difficult to demonstrate strain relatedness in an accurate and reproducible manner using available analytical methods. The need to track an organism and to cluster related strains are key elements in the effort to control human outbreaks of SE by identifying the sources of infections and prevent further exposures, e.g., by food recall procedures. Control of SE has been hampered by the low discriminatory potential of the available subtyping tests for sub-serovar classification such as phage typing and pulsed field gel electrophoresis (PFGE).

The explosive growth in massively parallel sequencing techniques fueled in part by cost affordability, coupled with the development and increasing expertise in the field and application of bioinformatics [20, 21], have created an unprecedented opportunity to further understand the biology of SE and to explore genomics-based solutions for outbreak investigations [22]. Despite a growing body of literature on the application of genomics and bioinformatics to organisms of the genus *Salmonella*, the serovar Enteritidis has until recently received little attention; only one finished SE genome is available in the public domain [23]. The recent addition to GenBank of 106 draft genomes mainly from US isolates, albeit predominantly belonging to a single PFGE type [5, 24] dramatically improves on the available Enteritidis genome data and now provides the resource to carry out a comprehensive comparison of isolates from different parts of the world. The comparison of two SE isolates belonging to the same phage type (PT13), but different PFGE types demonstrated diversity of SE isolates at the level of single nucleotide polymorphisms or SNPs [25].

In our study, we used massively parallel sequencing and bioinformatics tools to sequence and characterize the chromosomes of SE strains of different PFGE and phage types isolated from poultry environments and potential food sources that did not enter the food chain in Canada. Analyses of the genomes and comparison to the reference strain SE P125109, an isolate from the United Kingdom, confirmed the similarities among different isolates of SE and reinforced the organism’s clonal nature. Simultaneously, we demonstrated inherent diversity among a number of SE isolates at many SNP loci and in their prophage content, paving the way for the development of tests that could be used to differentiate lineages and to subtype isolates of SE for the purpose of tracking through biological systems including foods, the environment and infected humans.

## Results

### Genomic DNA sequencing

Whole genome sequencing was carried out on genomic DNA samples obtained from a total of 11 SE isolates using either the Illumina or Roche 454 pyrosequencing platform (Table 1). The average number of bases per genome sequenced with the Illumina platform was 1,614,261,640 ± 136,182,564 (i.e., mean ± standard deviation; n = 5) and 146,560,275 ± 30,940,841 for the 454 Roche platform (n = 6).

**Table 1:** **Whole genome analysis of the chromosome of** ***Salmonella*** **Enteritidis isolates of Canadian origin**

ISOLATE ID	SEQUENCING STRATEGY	GENOME ASSEMBLY STATISTICS (NG50 contig size)	GENOME SIZE (bp)	NUMBER OF GENES
SE 1	Illumina (HiSeq), mate paired	474,844	4,678,914	4,707
SE 2	Illumina (HiSeq), mate paired	489,409	4,678,744	4,702
SE 3	Illumina (HiSeq), mate paired	728,910	4,679,131	4,702
SE 4	Illumina (HiSeq), mate paired	445,313	4,678,377	4,703
SE 5	Illumina (HiSeq), mate paired	478,671	4,678,571	4,703
SE 6	454 shot gun	125,874	4,677,619	4,712
SE 7	454, shot gun	150,082	4,710,936	4,736
SE 8	454, paired end	312,607	4,678,101	4,714
SE 9	454, paired end	225,654	4,671,261	4,684
SE 10	454, paired end	263,672	4,709,890	4,745
SE 11	454, paired end	290,941	4,702,741	4,729

Whole genome assembly of *Salmonella* Enteritidis chromosome was carried out from raw reads obtained from Illumina HiSeq or Roche 454 next generation sequencing platforms using a hybrid method consisting of *de novo* and reference assembly aided by genome mapping (see under Materials and Methods). Gene annotation of the finished genomes was carried out using xBASE, BaSys and RAST annotation programs.

### *Salmonella*Enteritidis genome assembly

**Figure 1:**
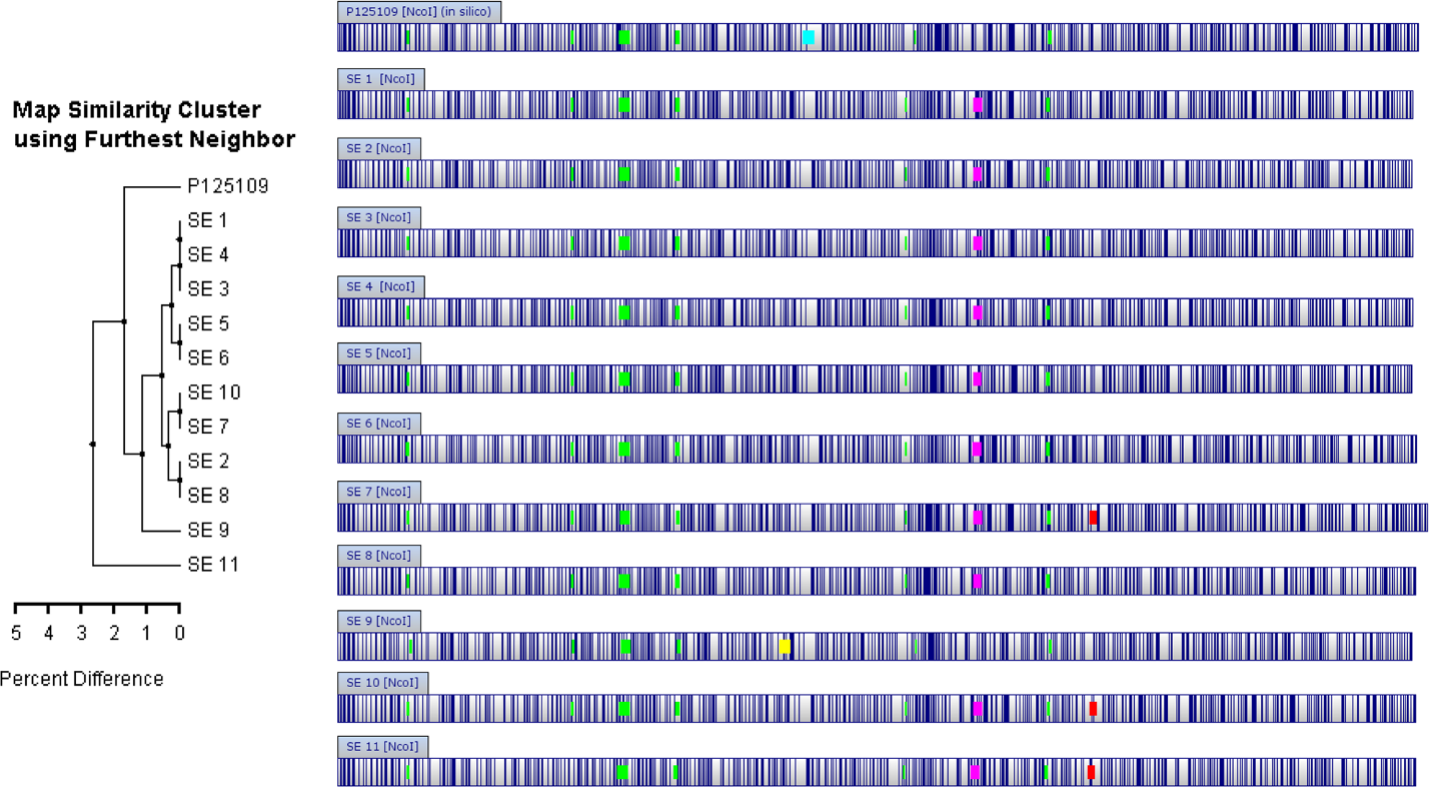
**Genome maps of eleven Canadian isolates of** ***Salmonella*** **Enteritidis.** Genome maps of contiguous DNA fragments of *Salmonella* Enteritidis isolates (n = 11) obtained following digestion with *Nco I* restriction enzyme (OpGen Inc., Gaithersburg, MD) were compared to an *in silico* map of the reference P125109 strain obtained from the GenBank (Accession Number AM933172.1) and all showed high similarity. The two main types of genetic variations observed were single nucleotide polymorphism within the component of the genome shared by all isolates (core genome; also see Table 1) and presence of prophage sequences (accessory genome). Six prophage sequences consisting of 4.0, 8.0, 8.2, 11.5, 14.8 and 42.0 kb depicted on the maps as “Green Square Symbol” were present in the reference genome and all field isolates with the exception of SE 11 which was missing the 8.2 kb prophage. Previously described prophages φSE 10, φSE 12&12A, and φSE 14 (reference [26]) match the 8.2, 42.0, and 14.8 kb prophage sequences, respectively, both in location and gene content. The unique prophage sequence in the reference genome “Blue Square Symbol” is the P125109 phage and matches previously described φSE20 (reference [19]). A 28 kb prophage designated as OLF-10058 (3) is depicted as “Red Square Symbol” and was observed in three isolates, namely: SE 7, 10 and 11. The recently described 36 kb ELPHis prophage (reference [27]) depicted in the maps as “Pink Square Symbol”, was found in all Canadian field isolates except one, SE 9, which was obtained from a clam (Table 3). SE 9 had a unique prophage, termed OLF-10012 “Yellow Square Symbol”, which has not been previously reported as a full 43 kb prophage in *Salmonella* Enteritidis.

All 11 genomes of SE showed remarkable similarity following full assembly using a composite of template-dependent reference assembly, *de novo* assembly and comparison with a genome map. Initially, we employed the reference assembly procedure using the published genome of the reference P125109 strain because of the ease of use. A very high level of similarity, ≥ 99.5%, was observed among the assembled nucleotide sequences of the field isolates and reference strain (Additional file 1). Next, we developed genome maps for each isolate which consisted of an orderly arrangement of all *Nco* I restriction fragments in each genome in the correct orientation. A high degree of similarity was evident among the genome maps of all our 11 SE isolates (Figure 1). Comparison of the genome map of each isolate with the corresponding *in silico* map of the reference assembled molecule confirmed a high degree of agreement but also revealed clear differences. Six or seven contiguous map fragments (size range = 1.7 – 12.0 kb, approx.; total size = 36.2 kb) and 1 - 4 other non-contiguous fragments (size range = 4.0 – 14.0 kb, approx) present in the genome maps could not be located on the corresponding reference assembled genomes. At the same time, an average of 38 small-sized fragments (range = 24 - 47 fragments, n = 11) with variable sizes from 17 – 2,000 bp were dispersed within the reference assembled genomes but were absent in their corresponding genome maps. To further examine these discrepancies, an *in silico* map was generated using the published nucleotide sequence of the reference SE strain P125109 since its use as a template in the reference assembly of raw reads will inevitably influence the output. The reference P125109 strain was found to contain a DNA fragment of approximately 50 kb which was absent in all 11 genome maps and the 11 corresponding *in silico* maps generated from the reference assembled chromosomes (Figure 1). BLAST analysis identified the 50 kb sequence as coding for the P125109 phage which, based on the genome annotation, contained 49 open reading frames (see Additional file 2). Nevertheless, a comparison of the numbers and sizes of DNA fragments used to develop the genome map and the fragments in a reference assembled genome which has been translated into an *in silico* map, still showed a very high agreement: >95%. But a discrepancy of up to 5% of a genome is substantial and requires further resolution. As a first step in developing a very high quality assembly and to overcome the inevitable distortion that arises whenever a reference genome is used as a template for assembling raw reads of an unrelated or distantly related isolate, we opted to use our collection of *de novo* assembled contigs of each genome as the basis for creating a high resolution assembly aided by the use of the corresponding genome map.

The Illumina mate pair reads assembled into an average of 14 contigs per genome (10 -18 contigs; n = 5 genomes) and collectively covered approximately 99% (98.7 - 98.8%) of the corresponding genome maps. The next best assemblies were observed for the Roche 454 paired libraries which had an average of 40 contigs (36 - 45 contigs; n = 4 genomes) all of which assembled into a single chromosome covering almost the entire genome length (99.9%). The shot gun Roche 454 libraries covered 97.6% or 99.0% of the genome over 59 or 63 contigs, respectively (n = 2 genomes). The DNA fragments missing from the reference assembled molecules but present in the genome map were readily identified among the *de novo* assembled contigs developed using the same raw reads. Notable among the DNA fragments present in both the *de novo* assembled chromosome and the genome map, but absent in the reference assembled genome, was a contiguous stretch covering approximately 36 kb which was present in 10 of the 11 field isolates and was found to code for the ELPHis prophage (see under Discussion). A second unique DNA sequence of 28 kb was detected in three of our genomes (SE 7, 10 and 11). This prophage sequence has not been previously described for Enteritidis and is now provisionally designated as SE-OLF-10058(3) phage, after the laboratory identification number of one of the three genomes in which it was found. A third unique prophage sequence, 43 kb, was found in only one genome and has been provisionally designated as SE-OLF-10012 phage.

The ≤1% disagreement between the genome map and the corresponding set of *de novo* contigs consisted of two types of gaps: those located between contigs following scaffolding into a single molecule using Newbler assembler (Roche paired end) or following concatenation of contigs according to the order predicted by genome maps (Illumina and Roche shot gun), and those gaps present within contigs due to the inability of the assembly algorithm to determine an accurate order of sequence. The overlay of the reference assembled genome with alignment of *de novo* contigs and genome maps allowed the identified gaps to be resolved and filled with the corresponding parts of the reference assembly that did not show any discrepancy with the genome map. The gaps were due to sequences subsequently identified as repeating elements and were eventually localized on the genomes. Seven gaps were each attributed to sequences of about 4.4 - 5.4 kb which were found to code for the ribosomal DNA (rDNA). The raw reads for the rDNA sequences typically assembled into three or parts of three separate contigs each containing the coding sequences for the three ribosomal RNA subunits, i.e., 23S, 16S and 5S rRNA, all belonging to rRNA *rrn* operon, known to occur seven times in the chromosome of all members of the *Salmonella* genus [28]. A second set of two or three gaps, depending on the genome, was due to the sequence of an oxaloacetate decarboxylase gene. The third type of repetitive elements affecting genome assembly was due to the cytochrome c oxidase operon which occurred at least twice in each genome and consisted of many but variable numbers of *ccm* genes. Occasionally, a specific gene that has not been duplicated in the genome, e.g., *SseI*, failed to assemble into a bigger contig and in the case of one genome, a transfer RNA (tRNA-Lys) occupied a gap site. To verify our hybrid assembly strategy and ensure all identified gaps were closed, we sequenced a total of 20 amplicons covering inter- and intra-contig gaps in one of the genomes (SE 2). The amplicon sizes as estimated on an agarose gel were in agreement with the expected gap sizes in all cases (±50 bp). Sanger sequencing of the amplicons showed excellent agreement with the genome sequences derived from the use of reference assembled molecules as part of the hybrid assembly strategey. As the basis of developing a high resolution genome assembly, the *de novo* procedure was very successful as judged by NG50 values of >125,000 bp for each of the genomes (Table 1).

Apart from our use of genome maps [29] and additional Sanger sequencing to ensure the completeness of the genomes assembled using the hybrid approach as described above, we also compared the entire nucleotide sequence of one of our isolates with a sequence of the same isolate generated with the Pacific Biosciences single molecule sequencing protocol followed by error correction of the assembled long reads at the Genome Quebec Sequencing Centre, Montreal. The two molecules had 99.98% nucleotide match and a complete agreement of their *in silico* maps (Ogunremi et al., manuscript in preparation).

To estimate the genome coverage, raw nucleotide reads from both Illumina and 454 sequencing platforms were trimmed and filtered. Sequences that were overabundantly represented in the raw reads which would have inflated coverage estimates were removed. Sequences showing extremely high or extremely low coverage would have artificially altered the actual coverage estimate if included. Fully assembled genomes had a median size of 4,678,683 bp (range = 4,671,261 to 4,710,936 bp; Table 1). We estimated the genome coverage to be 205× for the Illumina reads (range 169 - 229; n = 5) and 30× for the 454 reads (26 - 44; n = 6).

### Gene composition

Genome annotation using xBASE, BaSys and RAST led to the identification of genes present in each genome (Additional file 2). The number of gene coding sequences ranged from 4,684 – 4,745 indicating about 1% variation among the SE genomes (Table 1; Additional file 1). The pan genome of SE was estimated to consist of 4,833 genes (n = 11 genomes) with the core genome comprising of 4,600 genes (95%) and just 233 genes (5%) defining the accessory genome. Prophage sequences constituted the majority of our accessory SE genome (141 of 233 genes or 61%) as determined by genome annotation software and confirmed by BLAST analysis (Additional file 2). Five prophages or prophage remnants of 4.0, 8.0, 11.5, 14.8 and 42.0 kb were found in all genomes as well as in the reference P125109 strain. A sixth prophage sequence of size 8.2 kb was absent in one of the field genomes (SE 11) but was present in all of the remaining genomes including the reference genome (Figure 1). Prophage diversity was observed in the variable number of prophages present among the isolates and in the sequence composition of prophages when present in multiple isolates (Additional file 2). Two novel prophage sequences were also identified. A 28kb prophage fragment, provisionally designated as OLF-10058 (3) was observed in three genomes, namely SE 7, 10 and 11 (Figure 1 and Additional file 2). The second unique prophage designated as SE OLF-10012 prophage is a 43 kb sequence found in the clam isolate (SE 9; Figure 1 and Additional file 2).

### Single nucleotide polymorphisms

**Figure 2:**
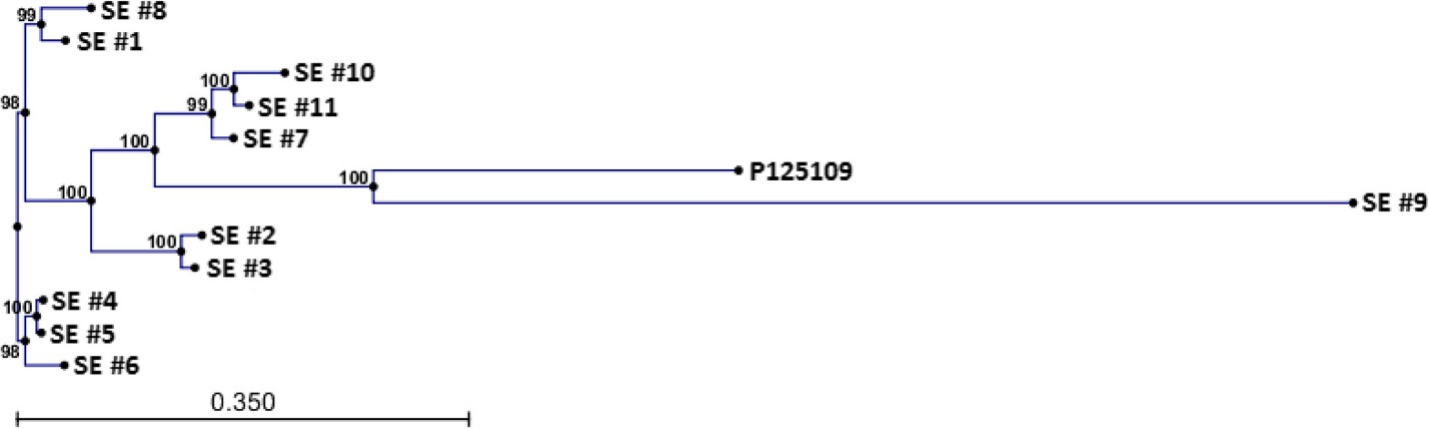
**Phylogenetic tree of** ***Salmonella*** **Enteritidis using single nucleotide polymorphic loci.** A total of 1,360 SNPs was used to assess the genetic distances among eleven Canadian field isolates of *Salmonella* Enteritidis and were compared to the reference P125109 strain. The reference strain obtained in the United Kingdom (reference [18]) and an isolate from a clam obtained as part of Canada’s Shellfish sanitation program (SE 9) were the most divergent of all genomes studied. SE 2 and 3 were obtained from the same poultry hatchery on the same day and showed genetic closeness based on SNPs and PFGE data although the phage types were different (Table 3). SE 7, 10 and 11 were obtained from the same premises and also showed close genetic distances by SNPs, PFGE and phage typing. SE 4, 5 and 6 showed genetic closeness and had similar PFGE results but SE 6 had a distinct phage result from the other two isolates. All three isolates were from the same province in Canada but SE 6 was obtained from a poultry premise different from where SE 4 and 5 originated.

A total of 1,360 SNPs were identified in the core genome of SE by comparing our group of eleven genomes with the genome of the reference P125109 strain (Additional file 3). Polymorphism occurred in both coding and non-coding regions. There was a preponderance of SNPs in genes coding for enzymes (27% of total number of SNPs). A phylogenetic tree constructed using all 1,360 SNPs showed a spatial relationship among the strains, and illustrated their genetic distances (Figure 2). A pairwise evaluation of the genomes indicated an estimated range of 23 - 905 SNPs among the genome pairs (Table 2).

**Table 2:** **Pairwise comparison of** ***Salmonella*** **Enteritidis genomes based on the number of single nucleotide polymorphic loci**

	SE 1	SE 2	SE 3	SE 4	SE 5	SE 6	SE 7	SE 8	SE 9	SE 10	SE 11
P125109	622	609	610	622	625	631	594	614	859	601	596
SE1		217	222	126	123	132	243	81	898	257	242
SE2			29	219	214	224	221	213	863	216	201
SE 3				234	221	225	224	211	864	217	202
SE 4					23	82	268	112	905	247	248
SE 5						75	261	111	894	252	237
SE 6							256	112	867	245	230
SE 7								248	866	49	38
SE 8									870	225	222
SE 9										857	840
SE 10											27

Loci showing single nucleotide changes in high resolution genomes of Canadian field isolates of *Salmonella* Enteritidis found using SNPsfinder software (http://snpsfinder.lanl.gov/) by comparison to the genome of the reference strain P125109 phage type 4.

### Evolutionary relationships among the SE isolates

Data from SNP analysis, gene content, PFGE and phage typing (Tables 1 and 3; Figure 2; Additional file 2) coupled with the evaluation of the accessory genome of each of our Canadian isolates (Figure 1; Additional file 2) allowed evolutionary inferences to be made in comparison to the reference P125109 strain. First, pairwise SNP analysis shows a clustering of the isolates based on their genetic distances. Thus, SE 2 and 3 appear to be closely related: each contains an identical number of genes (4,702 open reading frames, each) and differ by only 29 SNPs. This inference is supported by the source of the two isolates: they were obtained from the same poultry premise on the same day. The PFGE result also supported the similarity of the isolates although different phage typing results were obtained (PT 8 and PT 23; Table 3). Similarly, SE 4, 5 and 6 are closely related based on SNP pairwise comparison (≤82 SNPs), an observation supported by identical PFGE and phage typing results. These isolates were obtained from two poultry premises in the same Canadian province. Although all three isolates may well belong to the same biological clade, SNP analysis suggests a closer relationship between SE 4 and 5 (23 SNPs) than between SE 4 and 6 (82 SNPs) or SE 5 and 6 (75 SNPs). SE 9, which was obtained from a clam had the same phage type (i.e., result of first phage testing) and PFGE profile as three other isolates from poultry premises, i.e., SE 7, 10 and 11. On this basis, a close relationship could have been inferred but this conclusion would have been erroneous. Convincingly, the SNPs in the core SE genome and the prophage sequences both showed clearly that SE 9 was the most genetically distant of all isolates even when the reference strain from the United Kingdom is included in the comparison. SE 9 was lacking the ELPhis prophage found in all the other Canadian isolates, but instead had a unique prophage sequence that has not previously seen as a full prophage in SE until now (Figure 1).

**Table 3:** **Characteristics of eleven field isolates of** ***Salmonella*** **Enteritidis obtained in Canada and used in this study**

ISOLATE ID	YEAR OF ISOLATION	PROVINCE (SAMPLING LOCATION)	SOURCE	PHAGE TYPE	PFGE PATTERN
SE 1	2010	Quebec	Poultry hatchery environment	8	XAI.0003 BNI.0003
SE 2	2000	British Columbia	Poultry hatchery environment	8	XAI.0003 BNI.0009
SE 3	2000	British Columbia	Poultry hatchery environment	23	XAI.0003 BNI.0009
SE 4	2009	Quebec	Poultry hatchery environment	13a	XAI.0006 BNI.0007
SE 5	2008	Quebec	Poultry hatchery environment	13a	XAI.0006 BNI.0007
SE 6	2010	Quebec	Poultry hatchery environment	23	XAI.0006 BNI.0007
SE 7	2010	Ontario	Poultry hatchery environment	13	XAI.0038 BNI.0016
SE 8	2010	Alberta	Chicken	51	XAI.0007 BNI.0005
SE 9	2010	British Columbia	Manila clams	13 or 1b	XAI.0038 BNI.0016
SE 10	2010	Ontario	Poultry hatchery environment	13	XAI.0038 BNI.0016
SE 11	2010	Ontario	Poultry hatchery environment	13	XAI.0038 BNI.0016

Eleven isolates of *Salmonella* Enteritids were obtained from poultry environments (n = 9), a clam and a chicken in Canada were analyzed by phage typing using standard and Pulsed-field gel electrophoresis (PFGE; see details under Materials and Methods). Sample ID = Sample identification. Phage type results were inconsistent for SE 9 following re-testing.

## Discussion

We used two different sequencing platforms in this study, namely Illumina and Roche 454. The relative strengths and weaknesses of massively parallel sequencing platforms have been well described in numerous publications [21, 27]. In this study, both the Illumina and 454 platforms performed adequately well. The sizes of all eleven genomes were very similar all falling within a narrow range (mean ± SD) with no detectable size bias towards one sequencing platform or the other; our largest and smallest genomes, albeit not so disparate, were both sequenced with the Roche 454 platform. We, however, observed a significant difference in the ease of assembling depending on the type of library: paired end Roche 454 libraries were readily assembled into a single scaffold using the Newbler software. Raw reads from the Illumina’s mate pair or Roche 454 shot gun libraries were *de novo* assembled into multiple contigs following an initial effort and required further work to attain a single scaffold. Nevertheless, gaps of indeterminate nucleotides were still present in the single scaffold from the paired end 454 Roche sequence reads, thus requiring further analysis to achieve a finished genome by employing an innovative hybrid strategy to achieve high quality genome assembly.

Our effort to arrange contigs into a high quality assembly and resolve gaps present in the scaffolds benefitted greatly from the availability of genome (optical) maps. Knowledge of the order and orientation of *Nco* I fragments in each genome helped to confirm the arrangement of the contigs or scaffolds developed from the *de novo* assembly of raw reads. Predictably, a number of fragments found in the genome map were found missing in the reference assembled genome because of template bias. However, these fragments were found in the *de novo* assembled contigs. Indeed, *de novo* assembly by itself led to identification of approximately 99% of the chromosome of each of the 11 genomes studied while further comparison with the respective genome maps identified the approximate size of any gaps in these assemblies. The genome map also provided the needed confidence to employ parts of the reference assembled molecule to fill any missing gaps by using areas with identical patterns of *Nco I* restriction sites in the genome map and reference assembled molecule, a strategy that would otherwise have raised some doubts because of the bias inherent in reference assembled molecule. The general genetic similarity in the chromosome of SE and ability to generate good reference assemblies which, although may suffer the bias of the reference molecule, were nevertheless verified against optical maps and by Sanger sequencing and provided an advantage in facilitating the filling of gaps encountered during the course of producing finished genomes. We observed that a high number of genes were shared among our isolates, i.e., core genes at 95%, which was higher than estimates for *Salmonella* Paratyphi A (87.5%, n = 5 genomes [30], *Streptococcus agalactiae* (approximately 80%, n = 8 genomes; [31]), or *Listeria monocytogenes* (approx. 60%, n =28 genomes, [32]; 68%, n = 60 genomes [33]. Our genome annotation results indicate that the SE accessory genome pool consisted largely of prophages. The presence of a contiguous fragment in our genome map which was not present in the reference assembled genome led to further analysis including *de novo* assembly and the subsequent identification in 10 of 11 genomes of the recently described ELPHis prophage [34]. One of the significant observations in this study is how misleading and severely limiting is a sole reliance on reference assembled molecules to infer the genetic structure or to study diversity of any bacterial organism, even for an organism as clonal as SE. For example, we would have altogether missed the preponderance of the ELPHis prophage in our isolates if we relied only on a reference assembly strategy. Our hybrid assembly procedure was also useful in identifying a unique 28 kb fragment coding for bacteriophage P2 which was present in three of our field isolates (SE 7, 10 and 11; Figure 1 and Additional file 2) which we designate OLF-10058(3). This prophage, which has not been reported for the serovar Enteritidis, showed a high degree of identity (98%) with gp19 protein-containing phages present in each of *Salmonella* serovar Newport [35], serovar Hirschfeldii - formerly Paratyphi C [36], Paratyphi – formerly Paratyphi A [37], Heidelberg [38], and *Salmonella bongori* [39]. Significant matches (86-93% identity) in areas covering less than half the length of the phage were also observed with elements in non-*Salmonella* organisms such as *Escherichia coli* [26], *Enterobacter cloacae* [40] and *Cronobacter sakazakii* [41], but a unique contribution appeared to have come from serovar Typhimurium [42] with a high match (93%) which overlapped a majority of the phage sequence (18 kb of the 28 kb). Another unique prophage designated as SE OLF-10012 was found in the isolate obtained from a marine source (SE 9) and an intact copy of this prophage has not previously been reported in SE although as much as 7.6kb of the 43 kb prophage (17%) matched sequences annotated as prophage proteins in the SE reference P125109 strain [23]. A much larger portion of the prophage sequence (63%) had a significant match with *S. bongori* sequence which although largely un-annotated, contained an identified phage tail fibre sequence [39]. Our study suggests that variable prophage combinations may occur in SE strains thus providing an opportunity for subtyping SE. Many prophage encoded genes are transcriptionally silent, acquire mutations that convert them into pseudogenes and in time undergo degenerative changes and become phage remnants [43]. In addition, the persistence of prophage sequences in the genome even though they could contain different modules as a result of gene degradation in an isolate [44] could be exploited for lineage tracking. Collectively, these changes could be analyzed for the purpose of tracking a strain. We are currently pursuing a prophage-based subtyping procedure as an adjunct to a newly developed single nucleotide polymporphism-based (SNP-PCR) method (manuscript under preparation) which together should lead to a comprehensive description of the evolutionary map for SE in Canada. Isolates that are closest on our SNP-based phylogenetic tree (Figure 2) had identical or similar prophage sequence composition (Figure 1).

Single nucleotide polymorphism is one of the commonest forms of genetic variation and appears to be the most promising approach for the genotyping of a highly clonal organism such as SE. A recent study identified 247 chromosomal SNPs differentiating between two isolates of SE [17]. In our study, a comparison of 11 genomes with the reference SE strain led to the identification of 1,360 chromosomal SNPs. A phylogenetic tree constructed using these SNPs clearly demonstrated the genetic distances among the field isolates (Figure 2) despite what appeared to be a profound genetic similarity among them (Figure 1 and Tables 1 and 3). Results of pairwise SNP comparisons (Table 2) provided quantitative estimates of the genetic distances between 12 genomes analysis (11 + reference strain) as visualized by phylogenetic tree (Figure 2). The distribution of our pairwise SNP analysis showed that an isolate from clam was the most genetically distant of our isolates, and differed by an average of 876 SNPs (range 840 – 905) from all the Canadian isolates and the reference genome sourced from the United Kingdom. At the other end of the spectrum, we had two isolates that differed by just 23 SNPs inferring very close genetic similarity and confirmed by the historical metadata which showed that the isolates were obtained from the same poultry establishment one year apart (2008 and 2009). All the other pairwise comparisons occupied a range between 27 – 905 SNPs. The number of SNPs is expected to change as more SE genomes are studied. The distribution of our pairwise SNP count showed three discrete, non-overlapping populations, namely 23 - 134, 201 - 268 and 594 - 905 SNPs probably providing a quantitative estimate of isolates that are closely related, distantly related and unrelated, respectively. A note of caution is necessary. The use of bioinformatics software to infer characteristics of assembled genomes including the presence of SNPs usually require confirmation by other laboratory procedures before adoption for regulatory use because of a possible wide range of implications. Errors from the assembly procedure may translate to spurious SNPs. By the use of a rigorous genome assembly process aided by the availability of genome maps to confirm correct contig orientation, such errors have been minimized in this study. Furthermore, bioinformatics algorithms could have inherent errors which may not be easily obvious to a biologist. Confirmatory analysis either using other software or a wet laboratory approach could serve to detect such errors. All these mean that the use of bioinformatics analysis to deduce properties of a genome, especially at this stage of the development of the field of genomics will benefit from a wet chemistry approach especially for the development of a tool that is expected to serve a regulatory need. This approach is the basis of a separate communication in which we have used SNP-PCR to develop a highly discriminatory molecular subtyping tool for SE (Ogunremi et al., manuscript submitted).

## Conclusions

We have developed high resolution genome sequences for the chromosomes of eleven Canadian SE isolates by using a hybrid assembly method of sequence reads which relied on a composite of reference and *de novo* genome assemblies and comparison with genome maps. The procedure is easy to perform and allows the resolution of gaps that would have been caused by repetitive elements such as the *rrn* operon. High resolution assembly allows a definite assessment of the high degree of similarity among our field SE genomes and accurate description of the SE genome characteristics including the first report of the core and accessory genomes of SE based on fully assembled molecules. Despite its relatively large size, the core genome of SE shows abundant diversity expressed as SNPs which should allow for strain differentiation. We suggest that pairs of isolates that differ by < 150 SNPs may be closely related while isolates showing up to 600 SNPs or more are unrelated. The accessory genome of SE which consisted mostly of prophage sequences can also be exploited for SE lineage tracking because of variable composition of prophages. The same prophage sequence may show different degree of degeneration in distantly related or unrelated isolates. Whole genome analyses of SE isolates were useful in delineating and quantitatively estimating the genetic distances between isolates. This study has laid the ground work for further studies to develop a readily adoptable laboratory test for the subtyping of SE.

## Methods

### Strains of *Salmonella*serovar Enteritidis

Isolates of *Salmonella* serovar Enteritidis (n = 11) used in the study were retrieved from the Canadian Food Inspection Agency (CFIA) inventory. The isolates were all of Canadian origin and came from poultry environments (n = 9), a clam and a chicken (Table 3). Phage typing was carried out at the Public Health Agency of Canada (PHAC) Salmonella Reference Centre, Guelph as previously described [45]. Pulsed Field Gel Electrophoresis (PFGE) analysis was performed at the CFIA Ottawa Laboratory Fallowfield following an international, standardized protocol (http://www.pulsenetinternational.org/protocols/) involving the use of the restriction enzymes *Xba* I and *Bln* I to create signature molecular patterns which were electronically submitted to PulseNet Canada (National Microbiology Laboratory, PHAC, Winnipeg) for PFGE subtype designation.

### Genome sequencing

Genomic DNA was purified from SE culture using the Wizard^®^ Genomic DNA Purification Kit (Promega, Madison, WI). Frozen bacterial glycerol stocks were thawed, inoculated into BHI broth and then incubated at 37°C with agitation at 200 rpm. DNA purified from 1 ml of an overnight culture was assessed for quality by absorbance reading at optical density values of 260 and 280 nm (OD_260/280_) using a spectrophotometer (DU 730 Beckman Coulter, Mississauga, Canada), and was quantified by Quant-iT PicoGreen dsDNA Assay Kit (Life Technologies, Carlsbad, CA) with a fluorometer (VersaFluor, BioRad Laboratories, Mississauga, Ontario). Whole genome sequencing was performed at the Genome Quebec Sequencing Centre using the Illumina HiSeq 2000 platform (McGill University, Montreal) or at the Public Health Agency of Canada using the Roche 454 platform (National Microbiology Laboratory, Winnipeg).

The Illumina platform was used for isolates SE 1 – 5 (see Table 1) and the steps which consisted of mate pair library construction, DNA sequencing, and raw data processing were carried out according to the manufacturer’s protocol (Illumina, San Diego, CA). The remaining isolates were sequenced on the Roche 454 platforms using shot gun (SE 6-7) or paired end (SE 8 – 11; Table 1) library construction, DNA sequencing and raw read processing as recommended by manufacturer (Roche Diagnostics, GmbH, Mannheim, Germany).

### Genome sequence analysis and assembly

The assembly of each SE chromosome from raw genome reads into a single molecule was carried out by using a hybrid strategy that relied on *de novo* assembly, reference assembly and generation and analysis of genome (optical) maps. Raw reads from the Illumina genome sequencers were trimmed using the fastx toolkit and imported into the CLC Genomics Workbench (CLC bio, Aarhus, Denmark). Raw reads from the 454 Sequencer were processed and trimmed with the GS Reporter application software (Roche Diagnostics GmbH, Mannheim, Germany). *De novo* assemblies were generated using CLC Genomics (for Illumina and 454 shot gun sequences) or the GS *de novo* Newbler assembler (454 paired end sequences). A template-dependent or reference assembly version of each genome was also generated with the CLC Genomics software with the aid of the published chromosome sequence of SE strain P125109 phage type 4 [23]. For comparative purposes, other software or procedures were used to generate reference and *de novo* assemblies (e.g., Lasergene, DNASTAR Inc., Madison, WI; Geneious R6, BioMatters Limited, Auckland, New Zealand; Ray Assembler, Velvet assembler) for each genome (data not shown) but because the outputs were similar to that from CLC Genomics, the results from the latter software were used for further analysis. To assess the accuracy of the nucleotide reads in each contig, scaffold or whole molecule, and to confirm the orientations of contigs and their locations in the chromosome, all of the reference or *de novo* assemblies were aligned against a genome map generated for each respective chromosome using the Argus optical mapping system (OpGen Inc., Gaithersburg, MD) as previously described [29]. We generated independent maps for each of the eleven SE isolates by isolating high molecular weight genomic DNA from a single bacterial colony using the Argus Sample preparation Kit in conjunction with the Agencourt Genfind v2 DNA isolation kit as described by the manufacturer (OpGen Inc.) and ensured the DNA quality using the Argus QCard kit. After determining the choice of the *Nco* I restriction enzyme by means of the Argus Enzyme Chooser module (OpGen Inc.), stained genomic DNA was digested and the ensuing fragments were imaged using fluorescence microscopy on the Argus WGM system which automatically documents the fragment sizes in a manner that allowed an orderly arrangement as expected in the chromosome. The genome map was visualized using the MapSolver software (Opgen). Gaps and misalignment between the genome maps and the *in silico* maps generated from the nucleotide sequence of each candidate chromosome were identified by exploring the alignments of these regions with the corresponding reference assembled molecules generated with up to three different software programs (i.e., CLC Genomics, LaserGene or Geneious). The fragment judged to represent the correct fill for a gap was imported into Clone Manager (Professional edition, Scientific and Educational software, Cary, NC) and joined or concatenated with the genome using Clone manager or Geneious. In all instances, there were agreements between at least two sets of molecules produced by either reference or *de novo* assembly or genome mapping. The genome map was used as a standard to build up the genome scaffold from *de novo* assembled contigs however, there were occasions when small-sized DNA fragments, usually less than 1 kb, present as part of *de novo* contigs were observed either in the reference assembled genome or other *Salmonella* genomes developed during this study, but absent in the genome maps. To verify that all identified gaps were accurately filled by our hybrid assembly strategy, we developed PCR primers and amplified the fragment sequences spanning all the gaps identified in one of the genomes (SE 2). The sequence composition and size of each amplicon were determined by Sanger sequencing (ABI 3130; Life Technologies, Burlington, Ontario) and compared with the filled gaps which in all cases show full agreement with no detectable ambiguities (data not shown). *De novo* genome assembly was evaluated by measuring the NG50, the median contig length at which 50% of the total genome length has been covered. Contig lengths > 1000 bp were used for assembly evaluation. The success of the hybrid strategy of assembly was assessed by comparing one of the hybrid assembled genomes (SE 2; Table 1) with a genome sequence for the same isolate generated using the Pacific Biosciences single molecule read platform (courtesy of Dr. Ken Dewar, McGill University-Genome Quebec Sequencing Centre, Montreal, Canada).

### Genome annotation

Assembled SE genomes were annotated with the open source xBASE bacterial genome annotation pipeline (http://www.xbase.ac.uk/) using the reference SE strain P125109. The xBASE pipeline predicts coding sequences (genes) using the Glimmer software [46], tRNA genes using tRNAScan-SE [47] and ribosomal genes with RNAmmer [48]. The annotation process involved a protein BLAST analysis (http://htpp://www.ncbi.nlm.nih.gov) of translated coding sequences of the target and reference genomes to obtain the best match as shown by the E-value probability index [49]. The complete list of coding sequences for each genome was exported into Excel (Microsoft) to create a database after sorting the genes using an in-house algorithm. To overcome the potential constraint of using a reference genome in the annotation procedure as required for the xBASE procedure, we used two other programs, namely, Bacterial Annotation System or BaSys ([50]; https://www.basys.ca/) and Rapid Annotation Subsystem Technology, RAST ([51]; http://rast.nmpdr.org/), neither of which required that a reference genome be identified to achieve annotation. The list of coding sequences determined for each genome represented an amalgamation of all open reading frames detected by all three annotation programs. We directly investigated the prophage sequences present in each of the genomes using the prophage finder software [52]. We identified all genes that were present in each of the 11 genomes (i.e., core genome) and compared the number of genes that were found to be absent in at least one of the genomes studied (n ≤ 10; accessory genome).

### Single nucleotide polymorphisms

Single nucleotide differences in the genomes compared to the reference SE strain P125109 were detected by means of the SNPsfinder program ([53]; http://snpsfinder.lanl.gov/, Los Alamos National Laboratory, NM). Pairwise comparisons of the genomes were also carried out as a measure of genetic distance between members of each pair. Mapped reads of the genomes were analyzed by means of CLC Genomics software to confirm the presence of SNPs and to evaluate whether an observed nucleotide difference could have been due to a sequencing error. In a number of cases, oligonucleotide primers were developed for the PCR amplification and sequencing of the fragment containing the SNPs.

### Phylogenetic analysis

The SNPs detected by the bioinformatics pipeline analysis were concatenated and used to construct a phylogenetic tree for all eleven genomes and the reference genome for the purpose of representing the spatial relationship and genetic relatedness among the eleven isolates when compared to each other and to the reference genome. The tree was generated by the neighbour joining distance method [54] applied to all SNPs observed in each genome using CLC Genomics software; 100 bootstrap replicates were used to evaluate the robustness of the predicted phylogeny.

### Availability of supporting data

Apart from the data set provided within the article and as additional files, sequence reads and genome assemblies supporting the results are available in the GenBank repository, deposited under the following Bioproject ID PRJNA256209. The 11 assembled genomes are deposited under accession numbers CP009083 – CP009093.

## Electronic supplementary material

Additional file 1: **Similarity of** ***Salmonella*** **Enteritidis genomes following template-dependent assembly of raw reads and conversion to** ***in silico*** **genome maps.** Reference assembled genomes of eleven field isolates of *Salmonella* Enteritidis (SE) of Canadian origin and published sequence of the P125109 phage type 4 reference SE isolate were converted to in silico maps by means of the MapSolver software and compared for genetic relatedness. (PPT 250 KB)

Additional file 2: **Gene composition of** ***Salmonella*** **Enteritidis chromosome.** Annotations of 11 Canadian field isolates of *Salmonella* Enteritidis (SE) were carried out using three different software, namely Xbase, BaSys and RAST (see under Methods). All the coding sequences identified by the annotation software were compiled and compared. Genes or putative coding sequences present in all field genomes were identified as core genome while those present in 10 or fewer genomes were identified as accessory genome. Annotation of the P125109 genome has been included for comparison but was not considered in calculating size of the core and accessory genomes of the Canadian isolates. (XLSX 3 MB)

Additional file 3: **Single nucleotide polymorphic loci in the genomes of Salmonella Enteritidis obtained in Canada.** Comprehensive list of 1, 360 SNPs in the genomes of *Salmonella* Enteritidis obtained in Canada in comparison to the reference P125109 strain by a bioinformatics approach using the SNPsfinder software (http://snpsfinder.lanl.gov/). The gene or intergenic (IGS) location of each SNP is identified and probable function indicated, if known. (XLSX 212 KB)

## Abbreviations

CFIA: Canadian Food Inspection Agency
ID: Identification
PCR: Polymerase chain reaction
PHAC: Public Health Agency of Canada
PFGE: Pulsed field gel electrophoresis
PT: Phage type
SE: *Salmonella* Enteritidis
SNP: Single nucleotide polymorphism.
