# Supplementary figures and images for: High resolution assembly and characterization of genomes of Canadian isolates of Salmonella Enteritidis
Source: BMC Genomics. 2014 Aug 25;15(1):713. doi: 10.1186/1471-2164-15-713 (PMC4165908; doi:10.1186/1471-2164-15-713)

## Slide 1
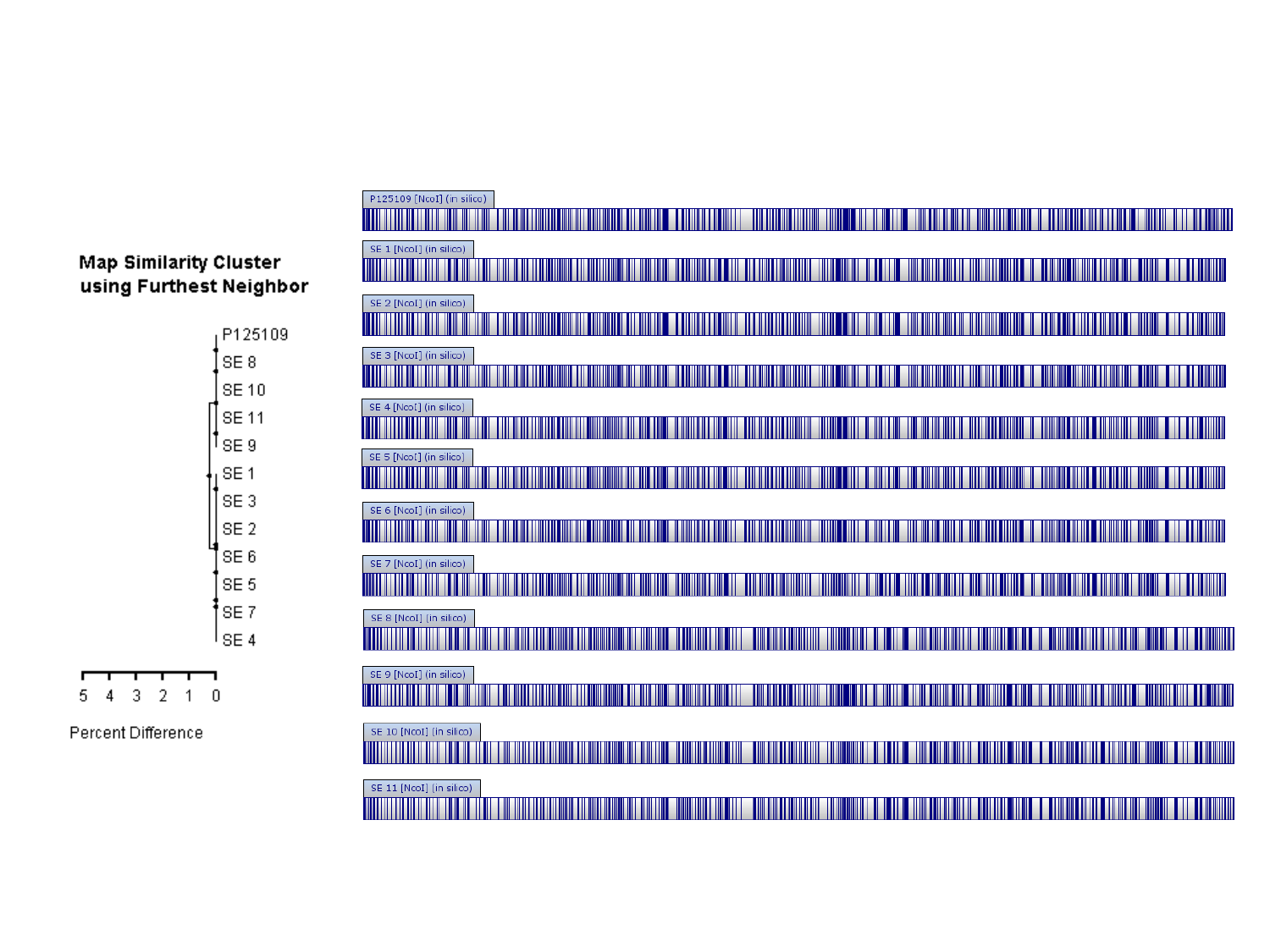

Supplement: Supplementary file 1 — Additional file 1: Similarity of Salmonella Enteritidis genomes following template-dependent assembly of raw reads and conversion to in silico genome maps. Reference assembled genomes of eleven field isolates of Salmonella Enteritidis (SE) of Canadian origin and published sequence of the P125109 phage type 4 reference SE isolate were converted to in silico maps by means of the MapSolver software and compared for genetic relatedness. (PPT 250 KB) [file 12864_2013_6436_MOESM1_ESM.ppt]
